# Dexmedetomidine Use in the Setting of Cocaine-Induced Hypertensive Emergency and Aortic Dissection: A Novel Indication

**DOI:** 10.1155/2011/174132

**Published:** 2011-09-26

**Authors:** Fahad Javed, Alexandre Miguel Benjo, Kiran Reddy, Muhammad Shoaib Akram, Shahzeb Afsar Khan, Manpreet Singh Sabharwal, Girish Nadkarni, Emad F. Aziz, Eyal Herzog

**Affiliations:** Division of Cardiology, St. Luke's Roosevelt Hospital Center, University Hospital for College of Physicians and Surgeons of Columbia University, 1090 Amsterdam Avenue, New York, NY 10025, USA

## Abstract

Aortic dissection is a potentially fatal but rare disease characterized by an aortic intimal tear with blood passing into the media creating a false lumen and with resultant high mortality depending on the location of dissection if not aggressively treated. Cocaine users are known to have a higher incidence of aortic dissection. We report here aortic dissection in a patient with cocaine abuse which did not respond to traditional medication regimes used currently in this setting. Worth mentioning is the use of an alpha-2 receptor selective agonist named Dexmedetomidine as a treatment modality to control hypertension in this patient, which is approved only for sedation of intubated and mechanically ventilated patients in the intensive care settings and for sedation during invasive procedures. This paper illustrates the practical beneficial role of Dexmedetomidine in controling blood pressure in the settings of cocaine-induced sympathetic surge when other treatment modalities fail.

## 1. Introduction

Aortic dissection is a potentially fatal but rare disease characterized by an aortic intimal tear with blood passing into the media creating a false lumen. Cocaine users are known to have a higher incidence of aortic dissection [1]. Aggressive blood pressure reduction is the paradigm of aortic dissection management [2, 3]. We present a case report of the use of dexmedetomidine, a selective alpha-2 agonist, as an antihypertensive and sedative agent in a female with acute aortic dissection after crack cocaine who was refractory to traditional interventions. 

## 2. Case Report

A 45-years-old woman brought in by EMS after she was found in distress on a subway platform. The patient with known history of diabetes type II and hypertension presented with stabbing chest pain radiating to the interscapular region after crack cocaine use. In addition to the chest pain and diaphoresis, the patient also complained of progressive pain and cold sensation over left lower extremity. Upon physical examination, the patient was noted to be agitated and combative with a blood pressure (BP) of 205/147 mmHg, a heart rate of 77 beats per minute, 17 respirations per minute, and was afebrile. Initial cardiovascular exam was positive for an S4 gallop and absent left lower extremity pulses. Lung examination was unremarkable. Initial management consisted of intravenous administration of a total of 9 mg of Lorazepam and 35 mg of Labetalol without acceptable reduction in BP. In addition to further Lorazepam, Labetolol later Esmololol infusions, IV Nitroglycerin was started and quickly titrated to 200 mcg/kg/hr but acceptable blood pressure reduction of blood pressure was still not achieved on this regimen (Table 1). A contrast-enhanced Computerized Tomography (CECT) of the thorax, abdomen, and pelvis confirmed a Stanford Type B aortic dissection beginning just beyond the left subclavian artery and extending to the iliac bifurcation. Upon arrival to the Cardiac Care Unit, decision was made to begin an infusion of Dexmedetomidine since all other therapies continued to be unsuccessful. The alpha 2 stimulation provided by the Dexmedetomidine was hypothesized to decrease the effects of the adrenergic surge induced by the cocaine thus mitigating alpha 1 and beta-receptor stimulation. Within 10 minutes of Dexmedetomidine bolus and continuous infusion, adjunct antihypertensive and sedative infusions were quickly weaned off. Evolving signs of ischemia in the left lower extremity warranted emergent femoral-femoral bypass. Vascular surgery team performed procedure successfully and uneventfully. Postoperatively BP control was maintained with Dexmedetomidine and Nitroglycerin then Nicardipine uneventfully. The total duration of Dexmedetomidine therapy was 42 hours. The patient was transitioned to oral antihypertensives simultaneously. The clinical course evolved without any further complications, and the patient was discharged after 14 days.

## 3. Discussion

Acute aortic dissection is a life-threatening disease with estimated incidence of 2.6 to 3.5 per 100,000 person-years [4–6]. The incidence is higher in certain populations including patients suffering from Marfan's syndrome and cocaine users [7]. Crack cocaine abuse accounts for up to 37 percent of dissections amongst select populations [8, 9]. Inhibition of norepinephrine reuptake by cocaine within peripheral sympathetic nerve synaptic clefts leads to a sympathetic surge within cutaneous and skeletal muscle beds [10–15]. The end result is an increase in peripheral vasoconstriction and tachycardia, which causes an increase in aortic shearing forces and in turn increases the risk for intimal rupture.

Intuitively, reduction of aortic shearing forces is a basic tenet of acute aortic dissection management. The stimulation of alpha-2 adrenergic receptors by Dexmedetomidine and Clonidine in the medullary vasomotor center reduces Norepinephrine turnover thus decreasing central sympathetic outflow. These pharmacologic interventions also increase parasympathetic outflow and inhibit sympathetic outflow from the locus coerulous which allows for increased stimulation of inhibitory neurons including the gamma amino butyric acid (GABA) system. GABA promotes sedation and analgesia, decreases minimum alveolar concentration of inhalational anesthetic needs, blunts sympathetic nervous response to noxious stimuli, and decreases blood pressure and heart rate [16–19]. Dexmedetomidine is FDA approved for sedation of intubated and mechanically ventilated patients in the intensive care setting and for sedation during invasive procedures. Recently, there have been several cases of “off label” use of Dexmedetomidine for patients who were found to fail traditional weaning procedures as the sedative effects of Dexmedetomidine have the benefit of not suppressing respiratory drive. Clonidine, another alpha-2 receptor agonist, and in some case reports Dexmedetomidine have been explored for cocaine and opiate withdrawal and detoxification [19–30]. There are also some experimental data demonstrating Clonidine's effect in blunting cocaine-induced hypertension [31]. In the same manner, there is one study that demonstrated that concomitant administration of Dexmedetomidine along with cocaine to healthy volunteers blunted its sympathomimetic effects; however, our search of the literature did not yield any reports of the clinical application of Dexmedetomidine specifically for cocaine-induced hypertension or even hypertension itself.

Compared to Clonidine, Dexmedetomidine has multiple advantages. Dexmedetomidine is 8 times more alpha-2 selective than Clonidine [32], has a shorter half-life (2-3 hours versus 12–24 hour), and is equally efficacious in Caucasians and African Americans; Clonidine has been shown to be less effective in African Americans [33]. These characteristics make Demedetomidine a suitable drug for the continuous intravenous infusion and a better alpha-2 adrenergic agent for the treatment of cocaine-induced hypertension. Furthermore, Dexmedetomidine may even have a role in noncocaine-induced hypertensive urgencies and emergencies or as an overall Clonidine substitute. However, some caution is advised in using Dexmedetomidine. Though it is a selective alpha-2 agonist, in higher doses it may result in hypertension due to alpha-1 stimulation that in the usual dose range is uncommon [34] and was not observed practically in our patient.

## 4. Conclusion

In conclusion, this paper demonstrates that Dexmedetomidine can be used in cocaine-induced hypertensive aortic dissection management secondary to its dual properties of reducing blood pressure and heart rate in addition to its sedative and analgesic properties which directly address cocaine-induced anxiety and dissection-related pain that further contribute to the adrenergic surge. This paper provides a practical insight that Dexmedetomidine can be considered as a therapeutic agent to address sympathomimetic consequences of acute cocaine ingestion and related hypertensive crisis, and possible other hypertensive situations though data is limited to date and needs further exploration.

## Figures and Tables

**Table 1:** 

Time PM	BP mm Hg	Nitroglycerine mcg/kg/hr	Esmolol mcg/kg/hr	Lorazepam	Labetalol mg/hr	Dexmedetomidine mcg/kg/hr	Sedation level
3:15	214/129	100	230	2 mg IVP	—	—	0
3:25		100	230	2 mg IVP	—	—	0
3:30	215/120	100	230	2 mg IVP	—	—	0
3:35		100	230	2 mg/hr	—	—	0
3:45	194/120	100	230	2 mg/hr	30	—	0
4:00	184/109	200	Off	4 mg/hr	60	—	0-1
4:15	164/94	200	Off	4 mg/hr	60	50 mcg bolus	1-2
4:30	109/52	Off	Off	4 mg/hr	Off	0.1	1-2
4:45	118/70	Off	Off	Off	Off	0.1	1-2
5:00	121/70	Off	Off	Off	Off	0.1	1-2
5:15	123/71	Off	Off	Off	Off	0.1	1-2
5:30	132/78	Off	Off	Off	Off	0.1	1-2
